# Synthesis, Characterization and *in Vitro* Antifungal Effect of Some Butyltin(IV) *N*-substituted 2-aminoethanethiolates

**DOI:** 10.1155/MBD.2001.165

**Published:** 2001

**Authors:** A. Smicka, V. Buchta, K. Handlir

**Affiliations:** 1 Department of General and Inorganic Chemistry Faculty of Chemical Technology University of Pardubice nam. Cs. Legii 565 Pardubice 532 10 Czech Republic; 2 Department of Biological and Medical Sciences Faculty of Pharmacy Charles University Heyrovskeho 1203 Hradec Kralove 500 05 Czech Republic

## Abstract

Six new *N*-substituted di- and tributyltin 2-aminoethanethiolates (cysteaminates) have been prepared and characterised by ^1^H, ^13^C and ^119^Sn NMR spectroscopy. All these compounds exhibit a good *in vitro* antifungal effect against selected types of human pathogenic fungi (*Candida albicans, Candida krusei, Candida tropicalis, Candida glabrata, Trichosporon beigelii, Aspergillus fumigatus, Absidia corymbifera, Trichophyton mentagrophytes*) and their activity is comparable with that of some antifungal drugs commonly used in the clinical use like ketoconazole. The structure-activity relationships in these compounds are discussed.


### SYNTHESIS, CHARACTERIZATION AND *IN VITRO* ANTIFUNGAL EFFECT OF SOME BUTYLTIN(IV) *N*-SUBSTITUTED 2-AMINOETHANETHIOLATES

A. Smicka*[1], V. Buchta[2] and K. Handlir[1]

[1] Department of General and Inorganic Chemistry, Faculty of Chemical Technology, University of Pardubice, nam. Cs. Legii 565, 532 10 Pardubice, Czech Republic <karel.handlir@upce.cz>
[2] Department of Biological and Medical Sciences, Faculty of Pharmacy, Charles University, Heyrovskeho 1203, 500 05 Hradec Kralove, Czech Republic <buchta@faf.cuni.cz>

**Abstract**

Six new *N*-substituted di- and tributyltin 2-aminoethanethiolates (cysteaminates) have been prepared and characterised by $^{1}H$, $^{13}C$ and $^{119}Sn$ NMR spectroscopy. All these compounds exhibit a good *in vitro* antifungal effect against selected types of human pathogenic fungi (*Candida albicans, Candida krusei, Candida tropicalis, Candida glabrata, Trichosporon beigelii, Aspergillus fumigatus, Absidia corymbifera, Trichophyton mentagrophytes*) and their activity is comparable with that of some antifungal drugs commonly used in the clinical use like ketoconazole. The structure-activity relationships in these compounds are discussed.

### Introduction

The organotin 2-aminoethanethiolates (cysteaminates) have been studied previously for their potential polydonor abilities.[1] However, biological properties, especially antifungal effects, of these compounds containing biologically interesting group $SCH_2CH_2N$ are almost unknown. It has been discovered recently that compounds of this type exhibit a relatively high *in vitro* antitumour activity[2]. Unfortunately, low solubility of these derivatives in water blocks further research in this context[3]. On the other hand, the lipophilic character of these derivatives seems to be no hamper for their antifungal effect. Thus, the set of new butyltin derivatives containing N-substituted group $SCH_2CH_2N$ has been prepared and their *in vitro* activity against some medically important yeasts (*Candida albicans, Candida krusei, Candida tropicalis, Candida glabrata, Trichosporon beigelii*) and molds (*Aspergillus fumigatus, Absidia corymbifera, Trichophyton mentagrophytes*) has been investigated.

### Results and discussion

The six new *N*-substituted organotin 2-aminoethanethiolates have been prepared: tributyltin-(2)-(1-piperidyl)ethylthiolate (**1**), tributyltin-2-(4-morfolinyl)ethylthiolate (**2**), tributyltin-2-(4-methylpiperazinyl) ethylthiolate (**3**) and dibutyltin-bis[2-(1-piperidyl)ethylthiolate] (**4**), dibutyltinbis[2-(4-morfolinyl) ethylthiolate] (**5**), dibutyltinbis[2-(4-methylpiperazinyl)ethyl-thiolate] (**6**). The general formula is given in Scheme 1.

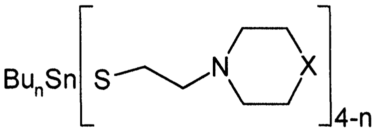

**1** n = 3; X = $-CH_2-$ **4** n = 2; X = $-CH_2-$

**2** n = 3; X = -O- **5** n = 2; X = -O-

**3** n = 3; X = $-NCH_3$ **6** n = 2; X = $-NCH_3$

Scheme 1

The studied compounds have been prepared by the reaction of tri- or dibutyltin chloride with corresponding *N*-substituted 2-aminoethanethiole in benzene solution in presence of natrium methoxide as a base. The preparations were carried out under anaerobic conditions. All prepared organotin compounds are colourless oils stable on air.

In the case of tributyltin derivatives (**1-3**) the values of $\delta(^{119}Sn)$ and $^{1}J(^{119}Sn,^{13}C)$ are about 80 ppm and 330 Hz, respectively(s. Tab.**IV**.); the C-Sn-C angles, estimated[4,5] from these values of coupling constants are $108^0$. These values indicate the presence of tetrahedrally bonded tin atom in the molecule, without any significant interaction between tin and nitrogen atom[4,5]. The values of $\delta(^{119}Sn)$ do not show any concentration dependence and thus intermolecular association seems to be excluded. This conclusion is in agreement with the results of previous study of $Bu_3SnSCH_2CH_2NEt_2$[1]. For dibutyltin compounds (**4-6**) – the values of $\delta(^{119}Sn)$ and $^1J(^{119}Sn,^{13}C)$ are about 130 ppm and 378 Hz, respectively (s.Tab.**IV**.), similar to these data for $Bu_2Sn(SCH_2CH_2NEt_2)_2$[1], therefore, we suppose analogous structural features in these compounds (four-coordinated tin atoms with C-Sn-C angle being $113^0$).

***In vitro* antifungal screening.**

The results of *in vitro* antifungal susceptibility testing against eight strains of potentially pathogenic fungi for humans are given in Table I as a **m**inimal **i**nhibitory **c**oncentration (MIC, in $\mu mol.l^{-1}$). For comparison, the MIC values of tri-, dibutyltinchloride, $Bu_3SnCl$ (**7**), $Bu_2SnCl_2$ (**8**), acyclic tributyltin-2-(*N*,*N*-dimethyl)aminoethanethiolate (**9**) and ketoconazole (**KTZ**) are given there, too.

The parent N-substituted thioles without organotin moiety did not show any activity against the fungal strains tested. On the other hand, the studied organotin derivatives showed good antifungal effects which were comparable with the *in vitro* activity of antifungal drugs like ketoconazole[6]. Tributyltin compounds studied exhibit interesting antifungal effect towards some strains, against which ketoconazole is markedly poorly active (e.g. **AF, AC**).

**Tab. I.** The MICs ($\mu mol.l^{-1}$) of studied compounds determined by microdilution broth test

| **Fungal strain** | **Compound** | | | | | | | | | |
|---|---|---|---|---|---|---|---|---|---|---|
| (incubation time) | **1** | **2** | **3** | **4** | **5** | **6** | **7** | **8** | **9** | **KTZ** |
| **TM** (72h) | 0.031 | 0.061 | 0.061 | 0.49 | 3.91 | 0.49 | 0.195 | 1.95 | 0.122 | 0.98 |
| (120h) | 0.061 | 0.061 | 0.122 | 1.95 | 3.91 | 0.98 | 0.195 | 1.95 | 0.24 | 1.95 |
| **CA** (24h) | 0.008 | 0.008 | 0.015 | 0.98 | 0.98 | 7.81 | 0.049 | 7.81 | 0.015 | 0.12 |
| (48h) | 0.49 | 0.49 | 0.24 | 3.91 | 1.95 | 15.63 | 0.049 | 15.63 | 0.24 | 0.12 |
| **CT** (24h) | 0.98 | 0.49 | 0.49 | 7.81 | 7.81 | 31.25 | 0.39 | 31.25 | 0.49 | 1.95 |
| (48h) | 0.98 | 1.95 | 0.98 | 15.63 | 7.81 | 62.50 | 0.78 | 62.50 | 0.98 | 3.91 |
| **CK** (24h) | 0.49 | 0.49 | 0.24 | 3.91 | 0.195 | 15.63 | 0.195 | 15.63 | 0.24 | 3.91 |
| (48h) | 0.98 | 0.49 | 0.49 | 3.91 | 0.39 | 15.63 | 0.195 | 15.63 | 0.49 | 3.91 |
| **CG** (24h) | 0.98 | 0.49 | 0.49 | 7.81 | 7.81 | 31.25 | 0.39 | 31.25 | 0.49 | 0.24 |
| (48h) | 1.95 | 1.95 | 0.98 | 15.63 | 15.63 | 62.50 | 0.78 | 62.50 | 1.98 | 0.98 |
| **TB** (24h) | 1.95 | 1.95 | 1.95 | 7.81 | 7.81 | 15.63 | 0.78 | 31.25 | 0.98 | 0.12 |
| (48h) | 3.91 | 7.81 | 3.91 | 15.63 | 15.63 | 31.25 | 1.563 | 62.50 | 1.95 | 0.24 |
| **AF** (24h) | 0.122 | 1.95 | 0.24 | 3.91 | 1.95 | 15.63 | 0.098 | 7.81 | 0.24 | 15.63 |
| (48h) | 1.95 | 1.95 | 0.98 | 7.81 | 7.81 | 15.63 | 0.39 | 15.63 | 0.98 | 15.63 |
| **AC** (24h) | 0.49 | 0.49 | 0.24 | 3.91 | 0.195 | 15.63 | 0.098 | 15.63 | 0.24 | 31.25 |
| (48h) | 0.98 | 0.49 | 0.49 | 7.81 | 0.195 | 31.25 | 0.098 | 15.63 | 0.49 | 31,25 |

**TM**: *Trichophyton mentagrophytes* 445, **CA**: *Candida albicans* ATCC 44859, **CT**: *Candida tropicalis* 156, **CK**: *Candida krusei* E28, **CG**: *Candida glabrata* 20/I, **TB**: *Trichosporon beigelii* 1188, **AF**: *Aspergillus fumigatus* 231, **AC**: *Absidia corymbifera* 272.

The data given in Table I reveal higher antifungal effects of the tributyltin compounds **1-3** than that of the dibutyltin analogues **4-6**. These results correspond with the dependence of antifungal and antibacterial activity of organotin compounds on the degree of tin atom alkylation in sequence $RSnX_3 < R_2SnX_2 < R_4Sn << R_3SnX$[7]. The organotin(IV) compounds with Sn-S bonding usually exhibit a considerable antifungal activity[8,9]. The studied N-substituted butyltin 2-aminoethanthiolates exhibit different activity in comparison with butyltin chlorides **7** and **8**. Thus, dibutylstannyl derivatives **4** and **5** are more active than dibutylstannyldichloride against seven of the eight fungal strains applied (except the mold **TM**). On the contrary, the tributylstannyl derivatives **1-3** are less active than tributylstannylchloride against six of the eight fungal strains applied. The derivatives **1-3** are more active than tributylstannylchloride only against the mold **TM** and the yeast **CA** (for one day incubation). These effects could be explained by different affinities of the studied compounds to water. An important role in the antifungal activity plays here the equilibrium between the lipophilic and hydrophilic character of the compounds during the transport through cell membranes[10].

The antifungal effect probably does not depend significantly on the structural changes of thiolate substituent. From the comparison with $Bu_3SnSCH_2CH_2N(CH_3)_2$ (**9**) it seems to be obvious, that the presence of heterocyclic arrangement itself is not important for the antifungal effect of this type of compounds. The S-

bonded substituent probably facilitates the transport through the cell membrane only, as it was already shown by *van der Kerk* and *Luijten*[11]. In the case of butyltin compounds, in which the substituent exhibits a biological effect as well, the synergic effect of substituent and butyltin group was observed[12].

### Experimental

*Syntheses*

Starting amines were commercially available and were dried by distillation with $CaH_2$. Thiirane was prepared from ethylene carbonate and KSCN[13]. Benzene was dried by refluxing with sodium under argon atmosphere and distilled before use. The preparations of sulfanylethylamines and butyltin derivatives were carried out under Ar atmosphere using standard Schlenk technique.

*N-(2-sulfanylethyl)-N'-methylpiperazine (X= -NCH₃)*: *N*-methylpiperazine, (4 g; 40 mmol) was dissolved in benzene (25 ml), than thiirane (2.4g; 40 mmol) dissolved in benzene (5 ml) was added in three portions in the intervals of 30 minutes at 60 °C. Then the mixture was stirred for 3 hours at 60°C and refluxed for 2 hours. The benzene was evaporated off under reduced pressure. Vacuum distillation (113-114 °C/1 kPa) of the residue afforded 4.4 g of colourless liquid (yield 68%).

The syntheses of other two 2-sulfanylethylamines were carried out using the same procedure with the following results:

*N-(2-sulfanylethyl)morpholine (X= -O-)*: b.p. 92-92,5 °C/1,1 kPa (yield 77 %).

*N-(2-sulfanylethyl)piperidine (X= -CH₂-)*: b.p. 69-70 °C/0,6 kPa (yield 64 %).

Synthesis of *tributyltin-2-(4-methylpiperazinyl)ethylthiolate*(**3**): *N*-(2-sulfanylethyl)-*N'*-methylpiperazine (1 g; 6.24 mmol) was dissolved in benzene (30 ml) and then the solution of $CH_3ONa$ (3.74 ml 1.67 M; 6.24 mmol) was added dropwise. A solution of $Bu_3SnCl$ (2.03 g; 6.24 mmol) in benzene (20 ml) was added and the reaction mixture was stirred at room temperature for 2 hours. The precipitate of NaCl was filtrated off and the solvent was evaporated under reduced pressure. Vacuum distillation (155-8 °C/18 Pa) of the residue afforded 1.91 g of a colourless liquid (yield 68 %).

The other butyltin compounds (**1**, **2**, **4-6**) were prepared using the analogous procedure.

Boiling points and the results of the elemental analysis of the studied compounds are given in Table II. The parameters of NMR spectra are given in Tables III and IV. The relevant marking of skeletal atoms is shown in Scheme 2.

**Tab. II.** The basic physicochemical and analytical parameters of the compounds **1-6.**

| compound | **1** | **2** | **3** | **4** | **5** | **6** |
|---|---|---|---|---|---|---|
| Boil. point [°C/Pa] | 120-2/12 | 115-20/5 | 155-8/18 | 165-7/8 | 172-4/7 | 183-5/2 |
| yield [%] | 66 | 51 | 68 | 71 | 61 | 60 |
| Element. analysis % | | | | | | |
| C (found/calc) | 52.94/52.55 | 49.52/49.56 | 51.03/50.79 | 50.33/50.68 | 45.69/45.72 | 47.86/47.92 |
| H (found/calc) | 9.50/9.52 | 9.10/9.01 | 9.39/9.42 | 8.81/8.89 | 8.12/8.06 | 8.85/8.77 |
| N (found/calc) | 3.10/3.23 | 3.15/3.21 | 6.18/6.23 | 5.41/5.37 | 5.26/5.33 | 10.00/10.16 |
| S (found/calc) | 7.48/7.38 | 7.29/7.35 | 7.08/7.14 | 12.36/12.30 | 11.96/12.21 | 11.49/11.63 |
| Sn (found/calc) | 27.35/27.33 | 27.28/27.21 | 26.48/26.42 | 22.83/22.76 | 22.50/22.59 | 21.59/21.52 |

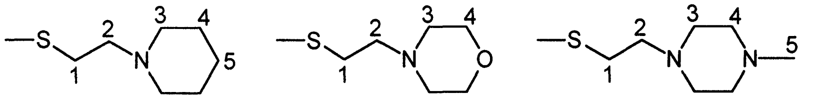

α β γ δ

$-CH_2CH_2CH_2CH_3$

Scheme 2

*NMR spectra*

$^1H$, $^{13}C$ and $^{119}Sn$ NMR spectra were acquired at 360.13, 90.56 and 134.28 MHz, respectively, on Bruker AMX 360 NMR spectrometer, using a 5mm tuneable broad band probe at 300K in $CDCl_3$ solutions. Chemical shifts are given with respect to $(CH_3)_4Si$ [$\delta(^1H)$ (HMDS) = 0.05ppm; $\delta(^{13}C)$ ($CDCl_3$) = 77.00 ppm] and $(CH_3)_4Sn$ [$\delta(^{119}Sn)$ = 0.0 ppm for $\Xi$ ($^{119}Sn$) = 37.2906174 MHz][14].

$^{13}C$ resonances were assigned on the basis of the values of $^nJ(^{119}Sn,^{13}C)$ coupling constants and standard $^{13}C$- APT techniques utilization in agreement with ref.[1].

$^1H$ resonances were assigned by means of expected spectral patterns, integral intensities and chemical shifts of non-bonded substituents[15].

**Tab. III.** The chemical shifts of the $^{1}H$ NMR resonances [ppm] of compounds **1-6**.

| $\delta(^{1}H)$ | **1** | **2** | **3** | **4** | **5** | **6** |
|---|---|---|---|---|---|---|
| α | 1.22 | 1.13 | 1.12 | 1.32 | 1.34 | 1.34-1.64 |
| β | 1.58 | 1.56 | 1.56 | 1.32-1.50 | 1.62 | 1.34-1.64 |
| γ | 1.32 | 1.32 | 1.32 | 1.32-1.50 | 1.41 | 1.34-1.64 |
| δ | 0.89 | 0.89 | 0.89 | 0.82 | 0.88 | 0.91 |
| 1 | 2.48 | 2.52 | 2.52 | 2.45 | 2.54 | 2.5-2.6 |
| 2 | 2.65 | 2.65 | 2.63 | 2.70 | 2.78 | 2.78 |
| 3 | 2.40 | 2.46 | 2.30-2.58 | 2.33 | 2.47 | 2.5-2.6 |
| 4 | 1.56 | 3.70 | 2.30-2.58 | 1.52 | 3.69 | 2.5-2.6 |
| 5 | 1.42 | - | 2.27 | 1.36 | - | 2.33 |

**Tab. IV.** The chemical shifts of the $^{13}C$ and $^{119}Sn$ NMR resonances [ppm] and $^{n}J(^{119}Sn,^{13}C)$ coupling constants [Hz] (in parentheses) of compounds **1-6**.

| | **1** | **2** | **3** | **4** | **5** | **6** |
|---|---|---|---|---|---|---|
| Sn | 79.7 | 80.5 | 80.2 | 126.3 | 130.6 | 129.2 |
| α | 13.3(330.14) | 13.3(330.57) | 13.3(329.45) | 17.9(378.70) | 18.0(377.31) | 18.2( - ) |
| β | 28.4(21.50) | 28.5(21.56) | 28.5(20.81) | 28.1(24.94) | 28.0(24.98) | 28.2(26.4) |
| γ | 26.9(61.04) | 26.9(61.04) | 26.9(61.04) | 26.6(74.9) | 26.5(72.48) | 26.6(76.3) |
| δ | 13.4( - ) | 13.5( - ) | 13.5( - ) | 13.3( - ) | 13.4( - ) | 13.5( - ) |
| 1 | 23.2 | 22.99 | 23.1 | 23.1 | 23.7 | 23.8 |
| 2 | 63.7 | 63.0 | 62.7 | 62.9 | 62.4 | 61.9 |
| 3 | 54.4 | 53.5 | 52.9 | 54.3 | 53.4 | 45.7 |
| 4 | 25.7 | 66.7 | 54.8 | 25.6 | 66.6 | 57.5 |
| 5 | 24.1 | - | 45.9 | 24.0 | - | 54.7 |

*In vitro antifungal testing.*

The *in vitro* antifungal testing was carried out by the modified microdilution broth format of the M27-A guidelines[16]. Quality control strains (*Candida albicans* ATCC 90028, *C. parapsilosis* ATCC 22019, *C. krusei* ATCC 6258) and ketoconazole (Janssen-Cilag, Beerse) as a reference drug were involved. All fungal strains were passaged on Sabouraud dextrose agar at 35°C prior to being tested.

Minimal inhibitory concentration (MIC) was determined by the following method: Dimethyl sulfoxide (DMSO) served as a diluent for all compounds tested. DMSO did not exceed the final concentration of 2 %. As a test medium was used RPMI 1640 (Sevapharma, Prague), supplemented with L-glutamine and buffered with 0.165 M morpholinepropanesulfonic acid (Serva) to pH 7.0 using 10 M NaOH. Each well of the microdilution tray was filled with 200 μl of the RPMI 1640 medium with a diluted compound tested and then inoculated with 10 μl of suspension of a given fungal strain in sterile water. Fungal inoculum was prepared to give a final size of 5 _ $10^{3} \pm 0.2$ CFU.ml$^{-1}$. The trays were incubated at 35°C and the MICs were visually read after 24 h and 48 h for all fungal strains tested except *T. mentagrophytes* (72 h and 120 h). The MIC was defined as at least a 80 % inhibition of the growth of control.

**Acknowledgements**

Authors thank to the Grant Agency of the Czech Republic (Grant No. 203/00/0920) and the Ministry of Education, Youth and Sport of the Czech Republic (COST 8.20 and MSM 111600002 programs) for financial support.

**References**

1. B. S.Saraswat, J. Mason, *Polyhedron(9),* **5** (1986), 1449.
2. M. Gielen, K. Handlír, M. Hollein, D. de Vos: *Metal-Based Drugs*, 7(5) (2000), 233-236.
3. G. Atassi, *Rev. Si Ge Sn Pb Cpds*, **8** (1985), 219.
4. J. Holecek, A. Lycka, *Inorg. Chim. Acta*, **118** (1986), L15.
5. J. Holecek, M. Nadvornik, K. Handlir, A. Lycka, *J. Organomet. Chem.*, **315** (1986), 299.
6. J. Van Cutsem, *Am. J. Med.*, **74**, Suppl.1B,. (1983), 9.; F.N Vincent-Ballereau, Patey O.N., Lafaix C., *Pharm. Weekbl. (Sci.)*, **13** (1991), 45.
7. B. D. James, S. Gioskos, S. Chandra, R. J. Magee, *J. Organomet. Chem.*, **436** (1992), 155. and references therein.

8. S.-G. Teoh, S.-H. Ang, S.-B. Teo, H.-K. Fun, K.-L. Khew, C.-W. Ong, *J. Chem. Soc., Dalton Trans.*, (1997), 465.
9. R. Barbieri, A. Silvestry, S. Filippeschi, M. Magistrelli, F. Huber, *Inorg. Chim. Acta*, **177** (1990), 141.
10. J. Susperregui, M. Bayle, G. Lain, C. Giroud, T. Baltz, G. Deleris, *Eur. J. Med. Chem.*, **34** (1999), 617.
11. G. J. M. van der Kerk, J. G. A. Luijten, *J. Appl. Chem.*, **4** (1954) 314; *idem, ibid.*, **6** (1956), 56.
12. J. J. Bonire, G. A. Ayoko, P. F. Olurinola, J. O. Ehinmidu, N. S. N. Jalil, A. A. Omachi, *Met.-Based Drugs*, **5** (1998), 233.
13. S. Searles, E. F. Lutz, *J. Amer. Chem. Soc.*, **80** (1958), 3168.
14. J. Mason (ed.), Multinuclear NMR, Plenum Press, New York, 1987, 625.
15. R. M. Silverstein, G. C. Bassler, T. C. Morrill: Spectrometric Identification of Organic Compounds, Willey, Chichester, 4th edn., 1981.
16. National Committee for Clinical Laboratory Standards. Reference method for broth dilution antifungal susceptibility testing of yeasts: Approved Standard, NCCLS document M27-A, 771 E. Lancaster Avenue, Villanova, PA, 19085, **1997**.

**Received: October 30, 2000 – Accepted: November 30, 2000 – Accepted in publishable format: September 10, 2001**

